# Case of Typhlitis

**Published:** 1886-06

**Authors:** J. G. Swayne


### CASE OF TYPHLITIS. By J. G. Swayne, M.D. Lond.

On Monday, August 24th, 1885, I was requested to see Mast. G., a boy aged 13, who was said to be suffering from a severe bilious attack, which his father attributed to his eating a quantity of fruit on the previous Saturday

When I saw him he was lying curled up in the bed on his right side, and complaining of pain in the right iliac region. There had been some nausea and vomiting, with loss of appetite. The tongue was slightly furred; the pulse 92, temperature 100. On examining the abdomen, I found a good deal of tenderness over the cæcum, but not over the rest of the abdomen. There was also dulness on percussion over that part and the ascending colon, which appeared to be enlarged, apparently from fæcal accumulation, although the swelling did not feel so firm as it usually does in such cases. In accordance with this view of the case, I gave him a dose of castor oil by the mouth, and also an injection of turpentine and castor oil. The latter came away before I left the house, but only brought with it some small fæcal masses of good colour. I also ordered him a mixture containing ♏iij. of diluted hydrocyanic acid every six hours to relieve sickness; his diet to be principally milk, beef tea, and arrowroot, with no solids.

On the next day, August 25th, I found little or no improvement. He still complained of pain, though scarcely so severe as before; the pulse and temperature had slightly fallen, the tenderness on pressure was not abated, and he said he felt that there was "something in the bowel which he could not pass." The castor oil had taken no effect. I directed him to continue his mixture, and to have poultices applied over the right iliac region, and when I saw him in the evening I ordered him a grain of calomel and four grains of Dover's powder.

On the following day, August 26th, I found that he had had a restless night, and had been sick after the powder. The bowels had not been relieved ; the tenderness and pain in the region of the head of the colon were much the same. The pulse was 82 and temperature 99. As he had been so sick I gave him an injection of mutton broth, and ordered very little food to be given by the mouth ; to continue the hydrocyanic acid, and suck some ice occasionally; to have a turpentine stupe applied over the tender part. With the exception of dulness over the cæcum, the rest of the colon was remarkably resonant on percussion. Some pills, containing in each one-sixth grain of belladonna, were ordered, one every night and morning.

On the next day, August 27th, I found him apparently a shade better. The belladonna pill had somewhat relieved the pain, and he had passed a quieter night. He had more desire for food and scarcely any sickness : pulse 80, temperature just above normal. There had been no action of the bowels. To continue the pills.

On August 28th I found him slightly better as regards the tenderness. The temperature was slightly below 98. He had still a fair amount of strength, and could readily get out of bed to have his bowels moved, and took a fair amount of fluid nourishment. There was, however, a tendency to coldness of the extremities. To continue the medicines, and poultices, sprinkled with laudanum, over the right iliac region, and also beef-tea injections by rectum. As there had been two or three attempts to have the bowels moved, I ordered two pills containing two grains of calomel, two of ext. hyoscyami, and four of ext. of colocynth ; to be followed by an enema of warm water and olive oil in the morning.

When I called the next morning, August 29th, I found that these measures had produced no action of the bowels, but had given rise to some griping and inconvenience. In other respects there was no alteration of the symptoms.

During the night following, about 2 a.m., I was called up to him because sudden collapse had come on, and on my arrival I found that death had taken place about five minutes before.

I made a post-mortem examination the following day, August 31st, about thirty hours after death. My brother assisted me. I found the abdomen much distended and discoloured, decomposition having already commenced. On cutting into it about one and a half pint of turbid, semi-purulent fluid of a dirty yellow colour escaped. The colon and rectum were tolerably healthy in appearance, and though distended with gas, in other respects nearly empty. Though the appearances of peritonitis were not very general over the abdomen, the lower portions of the ileum, especially near the caput coli, were much inflamed; the inflammation appearing to have radiated from that point as a focus. The surface of the intestine was covered with red streaks, patches and flakes of brownish-yellow lymph ; all the folds being much agglutinated and adherent both to one another and to the abdominal walls and pelvic viscera adjacent. With much trouble I separated the adherent convolutions of ileum until I could get at the caput coli and appendix vermiformis.

On examining the latter, I found that it contained a small, solid, smooth, oval body about the size of a small pea; but its walls were so inflamed and softened that they gave way in my hand just below the spot where the body in question was impacted. I made a section of this body with a sharp knife. It was soft and easily cut, and of a dark brown colour. On looking at the section with a magnifying glass of low power, it was seen to present a laminated arrangement, something like what would be seen in a section of a mulberry calculus, with an outer coating of phosphates. Some small fragments were found to be insoluble both in boiling alcohol and in dilute nitric acid. I crushed some of these into a fine powder, mixed this with water, and looked at it under the microscope with a half-inch magnifier. No trace of crystals of any kind could be seen; but the powder appeared to be almost entirely amorphous, and of a yellowish-brown colour. Here and there some masses and nodules of a roundish or oval shape could be seen, apparently united together by very fine filaments, so as to resemble bunches of currants.

From the characteristics I have mentioned I am led to conclude that this "calculus"—if it may be so called—consisted, as in a case I shall presently mention, of hardened fæcal matter; but where and how it was originally formed it is of course impossible to say.

The points chiefly worthy of notice in this case are: 1st, the age of the patient; 2nd, the insidious character of the attack; 3rd, the treatment, and the possibility of relief by a surgical operation.

1st. As to the age of the patient. He was a lad who had just entered his teens, and had therefore reached the time of life when these attacks are most liable to occur. In 32 cases collected by Dr. Crisp, 5 were under 10, 13 between 10 and 20, 7 between 20 and 40, and 7

between 40 and 60. Of these 32 cases, 29 were males, and 3 only were females. The greater proclivity of the male sex to these attacks has also been noted by other observers, and is a curious fact which has yet to be explained.

2nd. As to the insidious character of the attack. The pulse and temperature gave no indication from day to day of any increased inflammatory action. At my first visit the pulse and temperature were at the highest point, the pulse being 92 and the temperature 100. On the following day they had both slightly fallen. On the third day the pulse was 82, and the temperature 99. On the next day the pulse was 80, and the temperature 98½. On the fifth day the pulse was about the same, and the temperature rather below 98, and so on until the next day, when he died. There was certainly some increase of tenderness as the case gradually progressed from bad to worse, but this was by no means well marked. His position in bed, where he lay curled up on his right side, was certainly characteristic of typhlitis; but yet he never assumed that dorsal decubitus with the knees drawn up which is so characteristic of peritonitis, and within two days of his death he got out of bed of his own accord to have an action of the bowels. Although there was considerable tenderness on pressure over the cæcum, there was very little over the rest of the abdomen; and yet all this time peritoneal inflammation of a subacute character was smouldering and gradually increasing, with scarcely any pyrexia at first, and at last with a distinct fall of temperature just before the fatal termination.

In most of the recorded cases of typhlitis resulting from foreign bodies in the appendix, suppuration and perforation of that part appear to precede the symptoms of peritonitis; but the case just related shows that this course of events is not invariable, and that fatal peritonitis may arise without any escape of pus or foreign bodies from the appendix vermiformis. A very interesting case is related by Mr. Charters Symonds in the *British Medical Journal* for May 16th, 1885, in which, in concert with the late Dr. Mahomed, he removed a calculus from the vermiform appendage for the relief of chronic typhlitis. The first attack lasted seven weeks. The patient "during the first week vomited everything, and his bowels were not opened for ten days. During the whole time there was great tenderness in the right iliac fossa." This attack took place in January, 1883. The inflammatory symptoms then subsided; but the patient noticed afterwards a hard lump in the right groin, about the size of a walnut. After repeated attacks of pain and tenderness in this swelling, he was admitted, on July 16th, 1883, into Guy's Hospital, under the care of Dr. Mahomed. On August 24th Mr. Symonds cut down on the tumour, which was just above Poupart's ligament, and removed the calculus without opening the peritoneum. The calculus "was oval in shape, smooth on the surface, and measured three-quarters of an inch long and rather more than half an inch wide. On section it showed a laminated calcareous capsule, containing hardened fæcal matter." It was still contained in the vermiform appendix. For three months after the operation the patient had a sinus at the seat of the operation; but this closed up, and he made a good recovery.

It will be observed that in this case, notwithstanding repeated attacks of inflammation around it, the concretion did not escape from the vermiform appendix; and as it could be plainly felt, the indications for surgical interference were clear. In my case, however, no distinct tumour could be felt during life, and the appendix was found to be much more deeply seated after death.

3rd. On reviewing the treatment of my own case with the light that was subsequently shed upon it by a post-mortem examination, it is a source of regret to me that I gave any aperients by the mouth, and that I did not keep the patient more continuously under the influence of opiates. If he had recovered from the first attack of peritonitis, as Mr. Symonds' patient did, it is just possible that he might have been cured by a subsequent surgical operation, provided suppuration had taken place around the appendix, and that the concretion had become sufficiently large to be recognised by the touch. But in the case I have related, the difficulties of diagnosis were great. The cæcum was covered by a mass of adherent folds of ileum, so that at the post-mortem examination it took me some time and trouble to separate and unfold these before I could get at the cæcal appendix. I am therefore led to the conclusion that the only chance of success in such a case as mine would be by performing the operation early, almost before the symptoms of peritonitis were fully developed—*i.e.* almost before I saw him,—and then the indications would not have been sufficiently clear to render such a proceeding justifiable.

